# Genotyping, Drug Susceptibility and Prevalence Survey of *Trichomonas vaginalis* among Women Attending Gynecology Clinics in Hamadan, Western Iran, in 2014–2015

**Published:** 2017

**Authors:** Mohammad MATINI, Hossein REZAEI, Mohammad FALLAH, Amir Hossein MAGHSOOD, Massoud SAIDIJAM, Tayebeh SHAMSI-EHSAN

**Affiliations:** 1. Dept. of Medical Parasitology and Mycology, School of Medicine, Hamadan University of Medical Sciences, Hamadan, Iran; 2. Dept. of Molecular Medicine and Genetics, School of Medicine, Hamadan University of Medical Sciences, Hamadan, Iran

**Keywords:** Genotype, Parasite, Prevalence, *Trichomonas vaginalis*, Iran

## Abstract

**Background:**

In spite of sufficient knowledge about phenotypic variation of *Trichomonas vaginalis*, its genetic characteristics are poorly understood. We carried out a molecular epidemiology study in which in vitro metronidazole susceptibility of *T. vaginalis* isolates was considered.

**Methods:**

This study was conducted on 862 women admitted to Gynecology Clinics in Hamadan, west of Iran, during 2014–2015. After recording the socio-demographic and clinical characteristics of participants, vaginal swab samples were taken and subjected to microscopic examination, culture, in vitro sensitivity testing and PCR-restriction fragment length polymorphism (RFLP) analysis.

**Results:**

*T. vaginalis* was detected in 1.9% (16/862) of the samples using two parasitological methods. The all *T. vaginalis* isolates that subjected to drug susceptibility analysis were sensitive to metronidazole with MICs ranged from 0.4 to 12.8 μg/ml. *T. vaginalis* genotyping by using actin gene and PCR-RFLP analysis identified three actin type; A (9, 56%), I (6, 38%) and E (1, 6%). No significant correlation was observed between actin genotypes and their clinical manifestation (*P*>0.05).

**Conclusion:**

The prevalence of *T. vaginalis* infection is not noticeable in the region and the most of isolates are hypersensitive to metronidazole. Further studies are needed to clarify the efficiency of the actin gene, as a reliable genetic marker, for molecular epidemiology of trichomoniasis.

## Introduction

*Trichomonas vaginalis* is a protozoan parasite caused trichomoniasis, a sexually transmitted disease (STD), worldwide. Although up to 50% of cases of *Trichomonas* infection in women is asymptomatic but it causes various complications such as adverse pregnancy outcomes, infertility, pelvic inflammatory disease and cervical neoplasia. Men with trichomoniasis are mainly asymptomatic and symptomatic women demonstrate a variety of clinical manifestations ranged from mild to severe infection (1–3). Trichomoniasis is the most widespread non-viral sexually transmitted infection (STI), more common than gonorrhea, chlamydia and syphilis infection, with an incidence of 276.4 million cases annually in the world (4). Recently, trichomoniasis is receiving more attention because of its role in enhancing both HIV transmission and acquisition. Symptomless of trichomoniasis has critical role in HIV transmission dynamics and associated with rising at least two-fold risk of HIV transmission, as well as trichomoniasis associated with increasing of HIV shedding up to four times (1–5, 6).

Treatment of trichomoniasis is countered with two important concerns including treatment failure and drug resistance to metronidazole. Centers for Disease Control and Prevention (CDC) has predicted that 2%–5% of clinical *T. vaginalis* isolates have some level of drug resistance to metronidazole, the only drugs of choice for cure of trichomoniasis (7).

Despite the global distribution and public health importance of trichomoniasis, there is a little information about causes of the various features of the infection and ambiguities about the pathogenicity, drug resistance and other epidemiological aspects of *T. vaginalis* have not been clarified. Researches based on genetic analysis of the parasite would be very useful to answer these questions. Several studies have been carried out to identify genetically the parasite by using different genetic markers and techniques that a few of them have been promising (8–13).

Because of this issue, molecular epidemiology of trichomoniasis was conducted, using actin gene and PCR-restriction fragment length polymorphism (PCR-RFLP) in women referring to outpatient clinics in Hamadan. Furthermore, in vitro metronidazole, susceptibility testing of *T. vaginalis* isolates was performed to increase our understanding of metronidazole therapy of trichomoniasis.

## Materials and Methods

### Patients, samples collection and procedures

Overall, 862 symptomatic and asymptomatic women, attending Gynecology Clinics in Hamadan, western Iran were enrolled during 2014–2015. The individuals received vaginal treatment or metronidazole therapy within the two weeks prior to the study was excluded. After receiving an informed consent from all participants, socio-demographic characteristics and clinical signs and symptoms of women such as vaginal discharge, itching, irritation, burning and frequent urination and dyspareunia were collected by interviewing. During vaginal examination, sampling was performed by three sterile cotton swabs and subjected to wet mount preparation, Dorset culture medium, and Gram-stained vaginal smear. They were transferred to the parasitology research laboratory of Hamadan University of Medical Sciences. Wet mount preparations were immediately made and culture medium samples were incubated at 35.5 °C surveyed daily until they turned positive or up to seven days as described previously (14).

### In vitro metronidazole sensitivity assays

The Minimum Inhibitory Concentration (MIC) is the lowest concentration of drug that causes all of the parasites to become immobile in the culture microtiter plate well scanned microscopically after 48 h incubation (15). Drug susceptibility assay was carried out according to the Meingassner method and CDC protocol (15, 16). Briefly, metronidazole powder (Sigma Chemical Co. St Louis) was dissolved in distilled water and sterilized by Millipore filters (0.22 μm) and then serial twofold metronidazole dilutions were prepared to range from 400 to 0.1 μg/ml by using Diamond’s medium. The cell parasites in logarithmic phase of growth axenically cultured applied for sensitivity testing in aerobic condition. Finally, after 48 h incubation, MICs of the parasites were determined accordance with the method previously described in detail (17).

### DNA extraction, PCR amplification, and RFLP analysis

After cultivation of isolates on Diamond’s medium, parasite cells were applied for total genomic DNA extraction by DNA extraction kit (Cinnagen, Iran), according to the manufacturer’s instructions. DNA purity and concentration was measured by NanoDrop 1000 spectrophotometer.

Nested PCR was performed by using outer primer, Tv8S (5′-TCTGGAATGGCTGAAGAAGACG-3′) and Tv9R (5′-CAGGGTACATCGTATTGGTC-3′), and inner primers, Tv10S (5′-CAGACACTCGTTATCG-3′) and Tv11R (5′-CGGTGAACGATGGATG-3′) chosen from a previous study (13). The outer and inner primers amplify two fragments of the actin gene approximately 1260 and 1100 bp, respectively.

The PCR reaction mixture consisted of 5 μl of 10× PCR amplification buffer, 3 mmol/l MgCl2, 20 pmol of the each primer, 0.2 mM dNTP mix, 2μl template DNA and 2.5 unit/reaction of *Taq* DNA polymerase (all Fermentas life Sciences). Then final volume adjusted to 50 μl by adding sterile distilled water.

PCR amplification was conducted in two stages, using Eppendorf Mastercycler gradient thermocycler, according to previously described protocol (13). Briefly, the first stage was started by 5 min of denaturation at 95 °C and following that, ten cycles consisted denaturation at 94 °C, 30 sec, annealing at 55 °C, 30 sec, and 3 min extension at 72 °C. The second stage was composed of 25 cycles with similar denaturation and annealing steps but extension step was longer, 5 sec more, in each cycle and the end, a final extension step at 72 °C was done for 7 min. Final PCR amplification product was evaluated by 1% (w/v) agarose gel electrophoresis in 1X TBE buffer, containing SYBR Safe DNA gel stain (Invitrogen, Germany), 10 μl/100 ml.

RFLP analysis was performed by three restriction enzyme, *HindII*, *Tru1I* and *RsaI* (Fermentas, Thermo Scientific, USA), according to the manufacturer’s instructions. After digestion at 37 °C for 4 h, restriction fragments were visualized by SYBR Safe stain on a 3% agarose gel electrophoresis.

### Statistical Data Analysis

Data obtained from the questionnaires and laboratory examinations were analyzed in SPSS (ver. 16) (Chicago, IL, USA) by using chi-squared test (χ^2^), Fisher exact test and descriptive statistics *(P-*value< 0.05).

## Results

### Frequencies

*T. vaginalis* was detected in 1.9% (16/862) of the samples using two parasitological methods. Direct wet mount was positive for 0.9% (8/862) and Dorset’s culture was positive for 1.6% (14/862) of the individual samples. The age of infected women with *T. vaginalis* was between 19 and 48 yr old and the highest frequency of infection was observed in the 30–39 yr age group (56.2%). Although, significant relationship was observed between trichomoniasis and education but relationship between trichomoniasis and age groups, residency, marital status and occupation were not statistically significant (Table 1).

**Table 1:** Prevalence of trichomoniasis among studied women by demographic characteristics

**Characteristics**	**Trichomoniasis Positive no(%)**	**Trichomoniasis Negative no(%)**	**Total no**	***P*-value**
**Age (Year)**				0.392
<20	1 (3.45)	28 (96.55)	29	
20–29	3 (1.23)	241 (98.77)	244	
30–39	9 (2.73)	321 (97.27)	330	
≥40	3 (1.16)	256 (98.84)	259	
**Education**				<0.001
Illiterate	0 (0.00)	133 (100.00)	133	
Primary	11 (6.32)	163 (93.68)	174	
Secondary	2 (1.09)	180 (98.91)	182	
High school	3 (0.94)	318 (99.06)	321	
College	0 (0.00)	52 (100.00)	52	
**Residency**				0.423
Urban	15 (1.98)	746 (98.02)	761	
Rural	1 (1.00)	100 (99.00)	101	
**Occupation**				0.29
Housewife	16 (2.00)	783 (98.00)	799	
Official job	0 (0.00)	63 (100.00)	63	

Two of the three pregnant participants who underwent vaginal examination suffered from trichomoniasis. The most frequent signs and symptoms of trichomoniasis were vaginal discharge (16, 100%), genital itching (12, 75%), irritation (10, 62.5%), dysuria (11, 68.7%) and dyspareunia (8, 50%).

### Metronidazole susceptibility

**Fig. 1:**
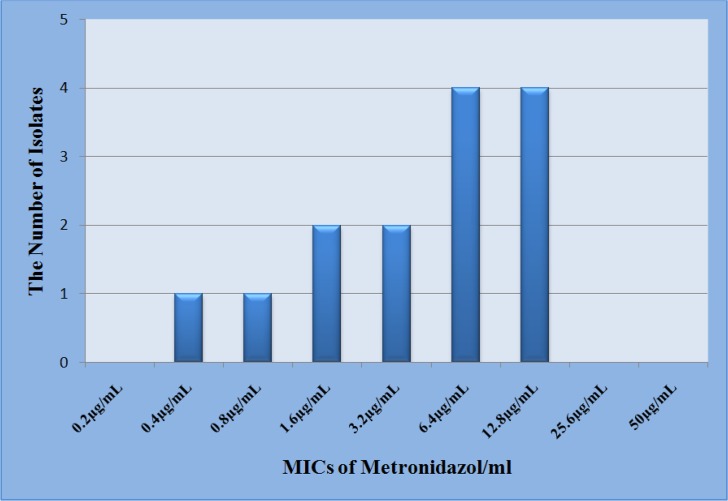
Distribution of metronidazole MICs of *T. vaginalis* isolated from studied women

Axenic culture of 14 *T. vaginalis* isolates was successful and they were subjected to drug susceptibility testing. All isolates were sensitive to metronidazole with MICs ranged from 0.4 to 12.8 μg/ml and the mean MICs ± standard deviation was 6.25 ± 4.78 (Fig. 1).

### Nested PCR and RFLP analysis

**Fig. 2:**
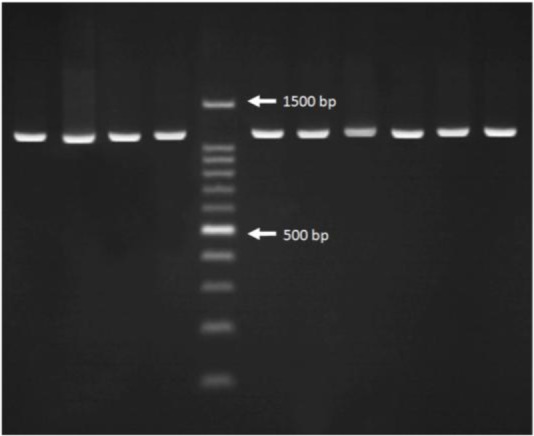
Agarose gel electrophoresis of nested PCR product amplification (1100 bp) from *T. vaginalis* isolates; DNA ladder: 100 bp

**Fig. 3:**
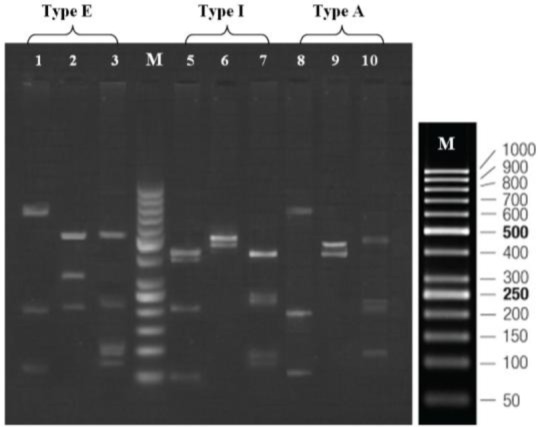
Representative RFLP patterns of the amplified 1100 bp fragment of actin gene obtained from *T. vaginalis* isolates. Lane 1, 5 and 8 digested by *HindII*; Lane 2, 6 and 9 digested by *Tru1*I; Lane 3, 7 and 10 digested by *RsaI*; M: DNA ladder (50 bp)

Nested-PCR assay amplified an anticipated fragment length of 1100 bp, 28 bp less than the open reading frame of the actin gene, belonged to the all *T. vaginalis* isolates (Fig. 2). Digestion of amplified nested-PCR products with *HindII* restriction enzyme led to two electrophoresis patterns including four (426, 401, 213, 60 bp) or three (827, 213 ,60 bp) DNA fragments of different lengths and classification of the parasites was done based on the presence of 827 bp or 426 bp and 401 bp of DNA fragments. Two other different patterns were obtained after digestion of the PCR products by *Tru1*I restriction enzyme, the first pattern with two DNA fragments of 581 and 519 bp and the second pattern with three DNA fragments of 581, 315 and 204 bp. The DNA fragment of 581 bp was common among all isolates. Finally, amplified PCR products were digested with *RsaI* restriction enzyme yielded three distinct electrophoresis patterns including a four-band pattern (568, 236, 190, 106 bp) and two different five-band patterns (568, 236, 106, 103, 87 bp and 452, 236, 190, 116, 106 bp) (Fig. 3).

The actin genotype of *T. vaginalis* isolates was determined based on combining the DNA fragment patterns (13) (Table 2). Given this method, 9 (56%) of the isolates belonged to genotype A, genotypes I and E were recovered in 6 (38%) and 1 (6%) of the parasites, respectively. The relation between genetic variability and clinical presentation of the *T. vaginalis* was investigated (18), and there was no statistical relationship between actin genotypes and clinical features of the isolates (*P*>0.05).

**Table 2:** Patterns of restriction enzyme digestion of amplified nested PCR products and determination of actin genotype of *T. vaginalis* isolates

***RsaI***									***Trul*I**					***HindII***						**Genotype**
**87**	**103**	**106**	**116**	**190**	**236**	**452**	**568**	**Pattern**	**204**	**315**	**519**	**581**	**Pattern**	**60**	**213**	**401**	**426**	**827**	**Pattern**	
−	−	+	−	+	+	−	+	1	−	−	+	+	1	+	+	−	−	+	1	**A**
+	+	+	−	−	+	−	+	2	+	+	−	+	2	+	+	−	−	+	1	**E**
−	−	+	+	+	+	+	−	3	−	−	+	+	1	+	+	+	+	−	2	**I**

## Discussion

Frequently, women with vaginitis were detected by vaginal discharge and itching or odor during their disease. Three different types of microbial agents frequently related to vaginal discharge in women are *Candida* species, bacterial species and *T. vaginalis,* a protozoan parasite (7). Extensive surveys and global estimation of burden of STIs are essential for strategic planning and appropriate interventions in the world.

In various regions of the world, the prevalence rate of trichomoniasis is different because of various epidemiological factors such as cultural and socio-economic status. In the United States, some studies indicated that 25% of referred to STDs clinics and 38% of African American women, persons belonging to vulnerable groups, were infected with *T. vaginalis* (2). Significant frequencies of the infection can be seen in some parts of Africa such as prevalence rate of 38% and 65% in Zaire (among HIV-positive women) and in a rural region in South Africa (in pregnant women), respectively (2). Lower the infection rates were reported from Islamic societies, for instance, ranged from 1.2% (Libya) to 28.1% (Saudi Arabia) (19, 20).

Different prevalence rates of trichomoniasis, similar to, but often lower than, the most reports from the other parts of the world, are observed in Iran. In recent years, the prevalence of trichomoniasis in different parts of the country was reported from as low as 0.9% (in women referred to a Gynaecology Clinic in Amol) (21) to as high as 10.2% (among women prisoners in Tehran Province) (22). Some other recent prevalence reports are available, including 2% in Kashan (23), 3.2% in Tehran (24), 2.2% in Hamadan (25) and 9.2% in Tabriz (26).

In the present study, frequency of trichomoniasis in women was 1.9% by using two parasitological methods, direct wet mount, and Dorset’s culture. Culture method missed two positive samples they identified by direct wet mount method. This may be due to that Dorset’s medium is less nutrient-rich medium than Diamond’s medium or may be due to other unknown biological factors. This prevalence rate is close to the two previous studies conducted in Hamadan in 2007 and 2010 with frequencies of 2.2% and 2.1%, respectively. In the past decade in the region, the infection rate of trichomoniasis has been constant in general population. Nearly, the status of *T. vaginalis* infection in Hamadan is similar to the most other parts of Iran, although some parts have significantly higher prevalence rate of the infection (26). Overall, difference in the results of the various studies is mainly due to different population groups studied and other effective factors such as having high-risk behavior, symptomatic or asymptomatic infection, sensitivity and specificity of diagnostic method and so on.

Variable phenotypic characteristics of *T. vaginalis* are usually observed in drug susceptibility, pathogenicity and clinical outcome of infection with the parasite. It is still unclear which genetic variability of the parasite or differences in host condition are more effective to impose diversifying. Molecular epidemiology and genetic-based research may present a new concept to address this issue. Some researchers employed random amplified polymorphic DNA (RAPD) assay. RAPD analysis of *T. vaginalis* was performed, using five random primers (OPD1-OPD5). This phylogenetic analysis showed some concordance between genetic relationship and metronidazole resistance and geographical origin of strains (8). Correlation between genetic variation and clinical features of *T. vaginalis* was investigated, using RAPD method with 10 random primers. In this study, four groups of infected individuals, in two categories, were distinguished; asymptomatic and symptomatic patients consist of mild, moderate, and severe trichomoniasis. An amplified fragment of 490 bp was found in the symptomatic group and this genetic marker was specific in the symptomatic patients (9). RAPD analysis of Iranian *T. vaginalis* isolates was showed no significant correlation between genetic diversity and origin of the parasites (27). Correlation between ribosomal intergenic spacer (IGS) polymorphism and clinical presentation of *T. vaginalis* was surveyed by using RFLP among 60 clinical isolates demonstrated no relatedness between IGS-RFLP patterns and symptomatology (28). Single Stranded Conformation Polymorphism (SSCP) analysis of ribosomal internal transcribed spacer (ITS) region was conducted on 50 Iranian *T. vaginalis* isolates yielded to distinct SSCP-PCR patterns. The polymorphism, due to a single point mutation at nucleotide position 66 (C/T) of the ITS1 region, was confirmed by nucleotide sequencing assay (29). In another research in Iran, actin gene of 50 *T. vaginalis* isolates was analyzed. This study showed seven-nucleotide sequence types based on SSCP banding patterns (30). Recently, microsatellite polymorphism and multilocus sequence typing assays were applied shown to be useful for genetic diversity and population structure analysis of *T. vaginalis* (11,12).

A promising study was conducted based on RFLP analysis of actin gene of *T. vaginalis* by using three restriction enzymes, *HindII*, *Tru1I,* and *RsaI*. In their study, 151 clinical isolates were collected from female sex workers, from Zambia and Kinshasa (the capital of Democratic Republic of Congo) and subjected to RFLP analysis. Eight actin genotypes were detected based on electrophoretic patterns of the restriction enzymes including genotype A (1.6%, 1.1%), E (55.7%, 5.5%), G (23%, 46.7%), H (9.8%, 16.7%), I (1.6%. 6.7%), M (3.2%, 0%), N (3.2%, 6.7%), P (0%. 5.6%), mixed (0%, 11%), non-classified (1.6%, 0%) in Kinshasa and Zambia, respectively (13).

In the present study, genetic characterization of *T. vaginalis* was carried out on symptomatic and asymptomatic infected women by using PCR of actin gene and RFLP analysis. Three actin genotypes were detected based on combined RFLP patterns (restricted groups) consist of genotype A (9, 56%), I (6, 38%), and E (1, 6%), without mixed genotype identification. This is contrary to another study (13) detected eight actin genotype with high frequency of genotype E, G, and low frequency of genotype A and I. One reason for this contradiction may be due to differences in the number and origin of examined samples and further studies in this area would help to elucidate these contrasts. The relationship between clinical presentation and genetic diversity of the isolates was investigated that no significant difference was found (*P*>0.05).

One of the other aims of this investigation was in vitro drug susceptibility of *T. vaginalis* isolates to metronidazole. In this regard, 14 axenic cultures of the isolates subjected to metronidazole susceptibility testing. The entire examined parasites were sensitive to metronidazole, the current drug of choice for treatment of trichomoniasis. Although according to CDC reports, low resistance to metronidazole (MIC equal to 50 μg/ml) can be detected in 2%–5% of clinical isolates and high resistance (MIC up to 400 μg/ml) may be rarely observed. In the previous study conducted on 50 clinical *T. vaginalis* isolates, collected from Tehran, Hamadan and Toyserkan city, one isolate was low resistant (MIC= 50 μg/ml) and two isolates had tolerance (MIC= 25 μg/ml) to metronidazole (17). In Iran, there is little-documented information available on drug resistance of the parasite and refractory trichomoniasis. Therefore, there is a great need for further studies in this regard.

## Conclusion

This study determined a rather low prevalence of *T. vaginalis* infection in the study population and probably other agents of vaginitis such as bacteria and fungi are more prevalent. In addition, actin analysis of *T. vaginalis* emphasized the differences in actin genotypes frequency based on geographical origin. Finally, more investigations must be done to evaluate the performance of these genetic markers in molecular epidemiology of trichomoniasis.

## References

[1] Secor WE Meites E Starr MC Workowski KA. Neglected parasitic infections in the United States: trichomoniasis. Am J Trop Med Hyg. 2014; 90 (5): 800– 4. 24808247 10.4269/ajtmh.13-0723 PMC4015567

[2] Schwebke JR Burgess D. Trichomoniasis. Clin Microbiol Rev. 2004; 17 (4): 794– 803. 15489349 10.1128/CMR.17.4.794-803.2004 PMC523559

[3] Petrin D Delgaty K Bhatt R Garber G. Clinical and microbiological aspects of *Trichomonas vaginalis*. Clin Microbiol Rev. 1998; 11 (2): 300– 17. 9564565 10.1128/cmr.11.2.300 PMC106834

[4] World Health Organization. Global incidence and prevalence of selected curable sexually transmitted infections-2008. WHO: 2012 http://www.who.int/reproductivehealth/publications/rtis/stisestimates/en/index.html

[5] Shafir SC Sorvillo FJ Smith L. Current issues and considerations regarding trichomoniasis and human immunodeficiency virus in African-Americans. Clin Microbiol Rev. 2009; 22 (1): 37– 45. 19136432 10.1128/CMR.00002-08 PMC2620632

[6] Wang CC McClelland RS Reilly M Overbaugh J Emery SR Mandaliya K The effect of treatment of vaginal infections on shedding of human immunodeficiency virus type1. J Infect Dis. 2001; 183 (7): 1017– 22. 11237825 10.1086/319287

[7] Workowski KA Berman SM. Centers for Disease Control and Prevention sexually transmitted disease treatment guidelines. Clin Infect Dis. 2011; 53 (suppl 3): S59– S63. 22080270 10.1093/cid/cir694

[8] Vanacova S Tachezy J Kulda J Flegr J. Characterization of trichomonad species and strains by PCR fingerprinting. J Eukaryot Microbiol. 1997; 44 (6): 545– 52. 9435127 10.1111/j.1550-7408.1997.tb05960.x

[9] Rojas L Fraga J Sariego I. Genetic variability between *Trichomonas vaginalis* isolates and correlation with clinical presentation. Infect Genet Evol. 2004; 4 (1): 53– 8. 15019590 10.1016/j.meegid.2003.12.003

[10] Stiles JK Shar PH Xue L Meade JC Lushbaugh WB Cleary JD Molecular typing of *Trichomonas vaginalis* isolates by HSP70 restriction fragment length polymorphism. Am J Trop Med Hyg. 2000; 62 (4): 441– 445. 11220758 10.4269/ajtmh.2000.62.441

[11] Conrad M Zubacova Z Dunn LA Upcroft J Sullivan SA Tachezy J Carlton JM. Microsatellite polymorphism in the sexually transmitted human pathogen *Trichomonas vaginalis* indicates a genetically diverse parasite. Mol Biochem Parasitol. 2011; 175 (1): 30– 8. 20813140 10.1016/j.molbiopara.2010.08.006 PMC2974001

[12] Cornelius DC Robinson DA Muzny CA Mena LA Aanensen DM Lushbaugh WB Genetic characterization of *Trichomonas vaginalis* isolates by use of multilocus sequence typing. J Clin Microbiol. 2012; 50 (10): 3293– 3300. 22855512 10.1128/JCM.00643-12 PMC3457461

[13] Crucitti T Abdellati S Van Dyck E Buve Molecular typing of the actin gene of *Trichomonas vaginalis* isolates by PCR-restriction fragment length polymorphism. Clin Microbiol Infect. 2008; 14 (9): 844– 52. 18844685 10.1111/j.1469-0691.2008.02034.x

[14] Matini M Rezaie S Mohebali M Maghsood A Rabiee S Fallah M Prevalence of Trichomonas vaginalis Infection in Hamadan City, Western Iran. Iran J Parasitol. 2012; 7 (2): 67– 72. PMC3469190 23109948

[15] Schwebke JR Barrientes FJ. Prevalence of *Trichomonas vaginalis* isolates with resistance to metronidazole and tinidazole. Antimicrob Agents Chemother. 2006; 50 (12): 4209– 10. 17000740 10.1128/AAC.00814-06 PMC1693974

[16] Meingassner J Havelec L Mieth H. Studies on strain sensitivity of *Trichomonas vaginalis* to metronidazole. Br J Vener Dis. 1978; 54 (2): 72– 6. 305807 10.1136/sti.54.2.72 PMC1046364

[17] Matini M Maghsood AH Mohebali M Rabiee S Fallah M Rezaie S In Vitro Susceptibility of Iranian Isolates of *Trichomonas vaginalis* to Metronidazole. Iran J Parasitol. 2016; 11(1: 46– 51. 27095968 PMC4835469

[18] Rojas L Fraga J Sariego I. Genetic variability between *Trichomonas vaginalis* isolates and correlation with clinical presentation. Infect Genet Evol. 2004; 4 (1): 53– 58. 15019590 10.1016/j.meegid.2003.12.003

[19] Kassem H Majoud O. Trichomoniasis among women with vaginal discharge in Benghazi city, Libya. J Egypt Soc Parasitol. 2006; 36 (3): 1007– 16. 17153709

[20] Madani TA. Sexually transmitted infections in Saudi Arabia. BMC Infect Dis. 2006; 6: 3. 16403220 10.1186/1471-2334-6-3 PMC1368987

[21] Valadkhani Z Assmar M Esfandiari B Amirkhani A Hassan N Lotfi ML Trichomoniasis in asymptomatic patients. Iran J Public Health. 2008; 37 (3): 113– 7.

[22] Valadkhani Z Assmar M Hassan N Aghighi Z Amirkhani A Kazemi F The prevalence of trichomoniasis in high-risk behavior women attending the clinics of tehran province penitentiaries. Iran J Med Sci. 2010; 35 (3): 190– 4.

[23] Arbabi M Fakhrieh Z Delavari M Abdoli A. Prevalence of *Trichomonas vaginalis* infection in Kashan city, Iran (2012–2013). Iran J Reprod Med. 2014; 12 (7): 507– 512. 25114674 PMC4126256

[24] Rezaeian M Vatanshenassan M Rezaie S Mohebali M Niromand N Niyyati M Prevalence of *Trichomonas vaginalis* using parasitological methods in Tehran. Iran J Parasitol. 2009; 4 (4): 43– 7.

[25] Rabieea S Fallah M Zahabic F. Frequency of Trichomoniasis in Patients Admitted To Outpatient Clinics in Hamadan (2007) and Relationship between Clinical Diagnosis and Laboratory Findings. J Res Health Sci. 2010; 10 (1): 31– 35. 22911914

[26] Gavgani A-SM Namazi A Ghazanchaei A Alizadeh S Sehhati F Rostamzadeh S Prevalence and risk factors of trichomoniasis among women in Tabriz. Iran J Clin Infect Dis. 2008; 3 (2): 67– 71.

[27] Valadkhani Z Kazemi F Hassan N Aghighi Z Esmaili I Talebi M. Gene Diversity of *Trichomonas vaginalis* Isolates. Iran J Parasitol. 2011; 6 (3): 101– 106. 22347304 PMC3279894

[28] Simões-Barbosa A Lobo TT Xavier J Carvalho SE Leornadecz E. *Trichomonas vaginalis*: intrastrain polymorphisms within the ribosomal intergenic spacer do not correlate with clinical presentation. Exp Parasitol. 2005; 110 (2): 108– 13. 15888292 10.1016/j.exppara.2004.12.012

[29] Matini M Rezaeian M Mohebali M Maghsood AH Rabiee S Rahimi-Foroushani A Genotyping of *Trichomonas vaginalis* isolates in Iran by using single stranded conformational polymorphism-PCR technique and internal transcribed spacer regions. Trop Biomed. 2012; 29 (4): 605– 12. 23202606

[30] Matini M Rezaie S Mohebali M Maghsood AH Rabiee S Fallah M Genetic Identification of *Trichomonas vaginalis* by Using the Actin Gene and Molecular Based Methods. Iran J Parasitol. 2014; 9 (3): 329– 35. 25678916 PMC4316563

